# A Case of Colchicine Overdose and Toxicity

**DOI:** 10.7759/cureus.85948

**Published:** 2025-06-13

**Authors:** Sneh Parekh, Ty J Merry, Vanshika Tripathi, Pramod Reddy

**Affiliations:** 1 Internal Medicine, University of Florida College of Medicine – Jacksonville, Jacksonville, USA

**Keywords:** acute rhabdomyolysis, alternative therapeutics, colchicine overdose, colchicine poisoning, colchicine toxicity, leukopenia - low white cell count

## Abstract

Colchicine is a commonly used medication that treats gout, familial Mediterranean fever, and pericarditis. Colchicine overdose is rarely seen; however, toxicity from colchicine overdose can have life-threatening consequences, precipitating multiorgan failure and death. Colchicine toxicity has a unique presentation that can result in severe abdominal pain, rhabdomyolysis, and leukopenia. Early identification of colchicine toxicity is necessary to help guide management, as no specific antidote exists. We present the case of a 31-year-old female who presented with severe abdominal pain and the sequelae of colchicine overdose, along with a discussion regarding her clinical course and management.

## Introduction

Colchicine is an antimitotic and alkaloid medication currently approved by the Food and Drug Administration (FDA) in the United States for the prophylaxis and treatment of gout flares in adults and the treatment of familial Mediterranean fever in patients aged four or older [1]. Off-label, colchicine has also been used most commonly for the treatment of acute and recurrent pericarditis and pseudogout, along with hepatic and primary biliary cirrhosis [1]. The mechanism of action of colchicine is through inhibition of β-tubulin polymerization into microtubules, thus disrupting cytoskeletal functions, while also preventing activation, degranulation, and migration of neutrophils [1]. Colchicine also acts as an antimitotic agent, disrupting mitotic spindle formation while blocking mitotic cells in metaphase [1,2]. At low concentrations, colchicine arrests microtubule growth and proliferation, and at high doses, it promotes microtubule depolymerization, which can ultimately lead to severe toxicity [2]. Tissue-specific adverse effects of toxicity can result in sensorimotor neuropathy, alopecia and rash, abdominal pain and diarrhea, pancytopenia, transaminitis and liver injury, and myopathy along with rhabdomyolysis [1].

Colchicine holds a very narrow therapeutic window of 1.2-2.4 mg/day in adults, along with a long elimination half-life of 26.6 to 31.2 hours [1,3]. As such, colchicine overdose is generally any ingestion exceeding this maximum dose of 2.4 mg/day [3]. Additionally, any ingestion exceeding 0.5-0.8 mg/kg of colchicine can be fatal [3]. The majority of deaths associated with colchicine overdose and toxicity tend to occur within seven to 10 days after ingestion [4]. Colchicine toxicity follows a phase-wise progression; Phase 1 of colchicine toxicity usually occurs within the first 24 hours post-ingestion and presents with gastrointestinal (GI) symptoms, including abdominal pain, vomiting, and diarrhea, due to the direct effect of inhibition of cell division on the rapidly dividing enterocytes within the GI tract, resulting in mucosal damage [3]. Phase 2 occurs within one to seven days after ingestion, with manifestations of metabolic acidosis, shock, myelosuppression, rhabdomyolysis, and multiorgan dysfunction of the kidneys, liver, and respiratory system due to the widespread inhibition of microtubule-dependent processes in these organ systems [3]. Finally, Phase 3 is often marked as the resolution stage, occurring from seven to 21 days post-ingestion, with hematopoietic recovery of leukopenia, along with resolution in electrolyte derangements that may be present [3].

Overall, colchicine toxicity is a relatively rare presentation, with a high risk for mortality. Here, we present the case of a 31-year-old female who presented with the sequelae of colchicine toxicity, ultimately recovering despite a high dose of ingestion, along with a discussion regarding the treatment and management of colchicine toxicity.

## Case presentation

**Figure 1:**
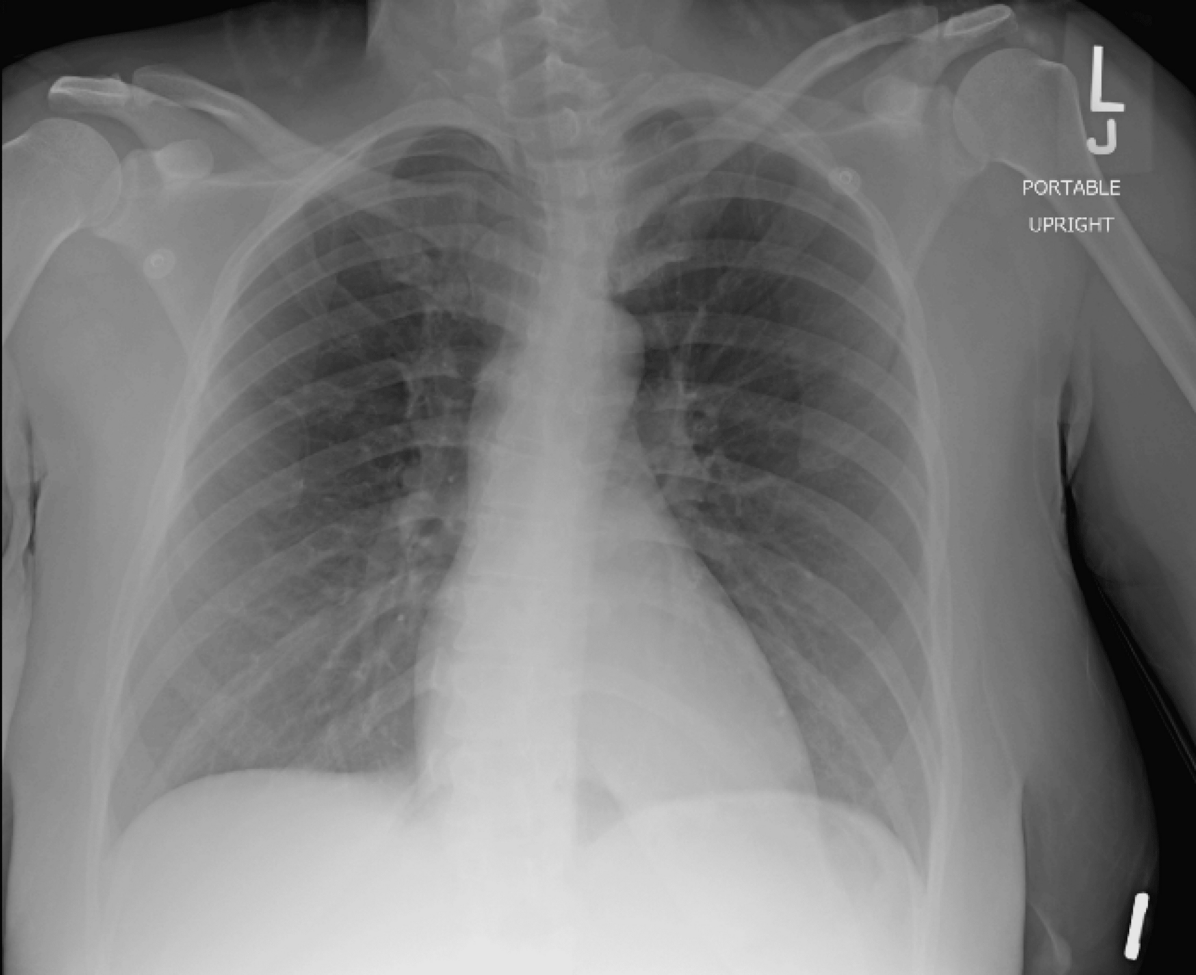
Chest X-ray obtained on admission was negative for any acute cardiopulmonary process

A 31-year-old female with a past medical history of asthma presented with diffuse abdominal pain, nausea, vomiting, diarrhea, and generalized weakness ten hours after ingestion of approximately 10-20 pills of 0.6 mg colchicine. She described the abdominal pain as "diffuse and colicky, with onset shortly after ingestion." On presentation, she was afebrile and hemodynamically stable, with a physical examination unremarkable apart from a non-distended abdomen, diffuse and non-localized abdominal pain both with and without light palpation, and hyperactive bowel sounds. Initial laboratory tests showed increased lactic acidosis (LA), a wide anion gap (AG), transaminitis, increased creatine kinase (CK), leukocytosis with an elevated white blood cell (WBC) count, and tests negative for coingestion of other toxic metabolic substances, including acetaminophen and alcohol. Imaging studies in the form of a chest X-ray were negative for any acute cardiopulmonary disease (Figure 1).

She received activated charcoal within 30 minutes of presentation and two liters of intravenous (IV) lactated Ringer's, and toxicology was consulted; thereafter, she was admitted to our internal medicine service.

Shortly after admission on day zero, she was upgraded to the intensive care unit (ICU) due to concern for impending multiorgan failure, with her LA peaking, as seen on day one in Table 1, while she continued to have persistent abdominal pain, along with a worsening wide AG metabolic acidosis and leukocytosis. Clinically, she did not display any other symptoms apart from the abdominal pain and nausea, and her vitals otherwise remained hemodynamically stable. After resolution of her LA, she was later downgraded back to our internal medicine service on hospital day two.

**Table 1:** Trend of WBC count, platelet count, lactic acid, creatine kinase, and AST/ALT values during hospitalization Of note, no labs were collected on day 10, the day of discharge. Additionally, note the rebound leukocytosis seen after administration of filgrastim and as the colchicine toxic effect runs its course. Those laboratory values indicated with a "---" were not trended daily after noted stabilization.

Test	Day 0 (admission)	Day 1	Day 2	Day 3	Day 4	Day 5	Day 6	Day 9 (one day prior to discharge)	Reference Ranges
Sodium (mmol/L)	142	134	134	133	131	132	133	129	135 – 145
Potassium (mmol/L)	4.0	3.7	3.4	4.0	4.1	3.6	4.0	4.3	3.4 – 4.5
Urea Nitrogen (mg/dL)	11	15	10	6	11	9	9	17	6 – 22
Creatinine (mg/dL)	0.88	1.28	0.86	0.66	0.59	0.61	0.61	0.76	0.51 – 0.96
Anion Gap (mmol/L)	20	16	13	12	12	15	16	13	4 - 16
WBC Count (x10^3^/UL)	15.43 (would later peak to 23.13)	16.99	6.17	2.04	0.96	6.48	23.29	13.69	4.5 - 11
Platelet Count (x10^3^/UL)	253	125	65	36	20	17	27	113	140 - 440
Lactic Acid (mmol/L)	4.8	5.1	1.7	---	---	---	---	---	0.7 - 2
Creatine Kinase (U/L)	426	1653	2563	2306	1394	645	373	---	22 - 195
Aspartate Aminotransferase (AST) (IU/L)	135	388	371	274	180	119	89	---	14 - 33
Alanine Aminotransferase (ALT) (IU/L)	36	50	41	44	68	84	114	---	10 - 42

For the rest of her hospital stay thereafter, she remained hemodynamically stable, with intermittent tachycardia, tachypnea, and hypertension, all likely related to her pain, as seen in Table 2. 

**Table 2:** Daily vital signs during hospitalization min: minute; mmHg: millimeters of mercury

Vital Signs	Day 0 (admission)	Day 1	Day 2	Day 3	Day 4	Day 5	Day 6	Day 10 (discharged)	Reference Ranges
Temperature (°C)	36.8	36.4	36.4	36.9	37.4	37.7	37.0	36.2	36.5 – 37.3
Heart Rate (beats/min)	91	95	92	87	68	70	114	107	60 - 100
Respiratory Rate (breaths/min)	17	34	31	27	18	18	15	18	12 - 20
Blood Pressure (mmHg)	138/103	119/86	109/76	127/77	144/89	171/105	167/114	121/83	90/60 – 120/80

Though she remained hemodynamically stable, her hospitalization was also complicated by persistent laboratory derangements, particularly with her sodium, her aspartate aminotransferase (AST) and alanine aminotransferase (ALT) values, CK, WBC counts, and platelet counts, as seen in Table 1.

Over the initial week following hospital admission, the patient began having diffuse myalgias secondary to rhabdomyolysis, with a CK level that would peak on hospital day two. Additionally, her clinical course also demonstrated a unique leukocytosis-to-leukopenia pattern, consistent with colchicine toxicity. While she had a significant leukocytosis on admission, by hospital day four, her WBC count fell to its nadir, significantly below the reference ranges of normal. Per our pharmacist's recommendations based on the indication of filgrastim in the treatment of neutropenia in acute radiation syndrome, which presents similarly with the destruction of cells with rapid turnover, she was administered filgrastim, a bone marrow stimulant directed toward the production of neutrophils. She was given subcutaneous filgrastim at a dose of 5 mcg/kg for a total of three days, after which her WBC count recovered. On hospital days four to five, the patient also became severely thrombocytopenic, with her platelet counts also falling to their nadir during the hospitalization. The thrombocytopenia eventually improved without medical intervention, suggesting the colchicine was clearing from her system. On hospital day 10, she was medically cleared and discharged from our service after noted stabilization and improvement in her laboratory value derangements, along with stabilization of vital signs, with resolution of her myalgias and abdominal pain, as seen in Tables 1, 2, respectively.

## Discussion

Colchicine toxicity is well-documented in the literature; however, specific cases of toxicity secondary to overdose are rare. There are no diagnostic tests to identify colchicine overdose, and so it remains a diagnosis of clinical suspicion. Colchicine’s toxicity is a combination of its dose-dependence, long elimination half-life, and mechanism of action [1,2,5]. High doses of colchicine, exceeding 0.5-0.8 mg/kg, its long elimination half-life of 26.6 to 31.2 hours, and its absolute bioavailability of 45% all provide a lethal combination leading to high plasma concentrations and slow elimination [1-3,5]. Colchicine's mechanism of action in binding to tubulin and disrupting microtubule proliferation, inflammasome inhibition, and interfering with the activation and migration of neutrophils leads to disruption of cell motility and mitotic arrest, which can ultimately result in multiorgan failure [1,2,5]. As such, the tissue-specific adverse effects of toxicity can result in sensorimotor neuropathy, alopecia and rash, abdominal pain and diarrhea, pancytopenia, transaminitis and liver injury, and myopathy along with rhabdomyolysis and renal failure [1].

Toxicity presents with GI manifestations, including severe abdominal pain and diarrhea, followed by multiorgan failure, shock, and sepsis due to a weakened immune system that often results in death [5]. If individuals survive the multiorgan failure stage, they will recover from the toxic ingestion, with recovery occurring within a few weeks after ingestion [5]. As such, clinicians need to maintain a high index of suspicion to correctly identify this toxidrome and appropriately manage patients.

With no antidote for colchicine toxicity, initial management involves administration of activated charcoal and supportive care. Activated charcoal is generally administered within the first hour after ingestion [3]. Though our patient presented to the hospital ten hours after initial ingestion, after which activated charcoal was administered, the benefits of administration still outweighed the risks with the goal of assisting in the elimination of the drug due to her toxic ingestion, the long half-life of colchicine, and her previously described clinical symptoms with laboratory abnormalities on presentation. Gastric lavage can also be performed; however, it is only effective within one hour of ingestion [6]. Some literature exists regarding the use of N-acetylcysteine (NAC) for the treatment of colchicine toxicity due to its ability as an antioxidant to scavenge free radicals; however, this data is limited, with two such successful cases of recovery from colchicine toxicity after administration of NAC [6,7].

In the management of colchicine toxicity, careful monitoring of clinical symptoms and laboratory values, including CK levels, liver enzymes, lactic acid, and cell counts, is crucial. No specific and formal guidelines exist specifying exact intervals regarding the serial monitoring of lab values. CK levels are generally measured daily, particularly in patients who present with elevated baseline levels on admission with associated complaints of myalgias and myopathy, until CK values trend down to near normal limits. Liver enzymes and cell counts are usually followed serially every eight to 24 hours, depending on the severity of transaminitis and abnormalities in cell counts, respectively, and one's overall clinical judgement. Lactic acid levels are generally followed every four to eight hours, particularly in the initial 24-48 hours following ingestion, with lactic acidosis a sign of severe toxicity, associated with poor outcomes, and one of the signs indicating the necessity for medical ICU admission, as in the case of our patient. Once lactic acid levels normalize, they are generally not followed, particularly with clinical signs of overall hemodynamic stability. As there is no specific and directed treatment once colchicine toxicity progresses into Phase 2, symptomatic and supportive management becomes crucial. This involves IV fluids for rhabdomyolysis, WBC stimulators, including filgrastim if patients become severely leukopenic, and an upgrade to an intensive level of care for closer monitoring and possible vasopressor support if multiorgan failure is impending. Additionally, no specific threshold values for intervention exist, and any patient ingesting toxic doses beyond those therapeutically prescribed should receive intervention [3]. With the help of our hospital’s toxicology department, there was early administration of activated charcoal upon presentation, despite being past the first hour following ingestion, and our patient was placed into an ICU level of care early on after admission to closely monitor clinical symptoms and laboratory values.

There also exists some research regarding colchicine-specific Fab fragments for therapy; however, the data is limited, and the efficacy in humans remains unproven. In one particular case, colchicine-specific Fab fragments were prepared from the antiserum of goats, which was then administered as an infusion to a critically ill patient secondary to colchicine overdose, resulting in significant clinical improvement [3,8]. Another study also showed how an engineered lipocalin (Lcn2) tightly complexes colchicine and can be used as an antidote to scavenge colchicine to reverse its toxic effects [3]. Nevertheless, these therapies have not become the standard of treatment due to the expense associated with Fab fragments, specifically, the need for more clinical studies of both therapies to determine their long-term effectiveness in humans, as they are not yet commercially available. Additional studies focusing on the efficacy of colchicine-specific Fab fragments and lipocalin, their long-term effectiveness and potential side effects in humans, and potentially other antidotes targeting toxicity may provide breakthroughs in the advancement of care for this toxic ingestion.

## Conclusions

Colchicine is an antimitotic agent, and overdose is a rare presentation that can often turn deadly. Colchicine toxicity presents with a phase-wise progression, and patients will present with GI symptoms first within the first 24 hours due to the direct toxic effect on rapidly dividing enterocytes. Thereafter, within one to seven days after ingestion, manifestations of rhabdomyolysis, pancytopenia, and multiorgan failure with dysfunction of the kidneys, liver, and respiratory system can present. Finally, if patients survive past these stages, resolution occurs from seven to 21 days post-ingestion, with hematopoietic recovery of leukopenia, along with improvement in electrolyte derangements and organ dysfunctions, results. Our patient presented with the classical signs of GI toxicity, rhabdomyolysis, pancytopenia, and liver injury. No formal guidelines specifying exact intervals for monitoring of laboratory values in colchicine toxicity exist. Management otherwise involves administration of activated charcoal as soon as possible upon identification of intoxication, ideally within the first hour after ingestion, with our patient receiving activated charcoal within 30 minutes of presentation, IV fluids, and close monitoring of laboratory values based on patient presentation and clinical judgement. These laboratory values include CK for rhabdomyolysis, AST/ALT for the associated risks of liver injury, lactic acid levels due to concern for ischemia and multiorgan failure, and WBC counts for the unique leukocytosis-to-leukopenia pattern similar to other toxicity patterns seen in literature. As no specific antidote exists for colchicine toxicity, supportive care with electrolyte replacement for derangements, filgrastim as a neutrophil stimulator, and close monitoring and treatment of the presenting symptoms are crucial. As such, this case aims to provide a framework for the unique presentation of colchicine toxicity, emphasizing the importance of treatments such as activated charcoal and filgrastim as an option for a neutrophil stimulator based on its use in acute radiation syndrome, along with providing a guideline for laboratory value monitoring.
